# Enhancing Autonomous Truck Navigation with Ultra-Wideband Technology in Industrial Environments

**DOI:** 10.3390/s24154988

**Published:** 2024-08-01

**Authors:** Pairoj Waiwanijchakij, Thanapat Chotsiri, Pisit Janpangngern, Chanchai Thongsopa, Thanaset Thosdeekoraphat, Nuchanart Santalunai, Samran Santalunai

**Affiliations:** 1 School of Electronic Engineering, Suranaree University of Technology, Nakhon Ratchasima 30000, Thailand; d6300265@g.sut.ac.th (P.W.); thanapat.c@groupmaker.co.th (T.C.); pisit.janpangngern@gmail.com (P.J.); chan@sut.ac.th (C.T.); thanaset@sut.ac.th (T.T.); 2 Department of Telecommunication Engineering, Faculty of Engineering and Technology, Rajamangala University of Technology Isan, Nakhon Ratchasima 30000, Thailand

**Keywords:** ultra-wideband (UWB) technology, autonomous vehicles, positioning technology, navigation systems, industrial automation

## Abstract

The integration of autonomous vehicles in industrial settings necessitates advanced positioning and navigation systems to ensure operational safety and efficiency. This study rigorously evaluates the application of Ultra-Wideband (UWB) technology in autonomous industrial trucks and compares its effectiveness with conventional systems such as Light Detection and Ranging (LiDAR), Global Positioning System (GPS), and cameras. Through comprehensive experiments conducted in a real factory environment, this study meticulously assesses the accuracy and reliability of UWB technology across various reference distances and under diverse environmental conditions. The findings reveal that UWB technology consistently achieves positioning accuracy within 0.2 cm 99% of the time, significantly surpassing the 10 cm and 5 cm accuracies of GPS and LiDAR, respectively. The exceptional performance of UWB, especially in environments afflicted by high metallic interference and non-line-of-sight conditions—where GPS and LiDAR’s efficacy decreased by 40% and 25%, respectively—highlights its potential to revolutionize the operational capabilities of autonomous trucks in industrial applications. This study underscores the robustness of UWB in maintaining high accuracy even in adverse conditions and illustrates its low power consumption and efficiency in multi-user scenarios without signal interference. This study not only confirms the superior capabilities of UWB technology but also contributes to the broader field of autonomous vehicle technology by highlighting the practical benefits and integration potential of UWB systems in complex and dynamic environments.

## 1. Introduction

The deployment of Autonomous Guided Vehicles (AGVs) across various sectors promises to revolutionize not only transportation but also industrial operations, offering unprecedented improvements in efficiency, safety, and cost reduction. As these technologies become increasingly prevalent, especially in high-stakes environments such as industrial logistics, the demand for precise and reliable navigation systems has become paramount. These systems must not only ensure the safety of operations but also enhance the efficacy and speed of tasks traditionally handled by human operators [1,2,3,4].

Among the array of positioning technologies, Ultra-Wideband (UWB) has emerged as a standout for its precision and resilience. UWB is a radio technology that uses very short-duration pulses covering a large portion of the radio spectrum. This attribute allows UWB to provide highly accurate location tracking, making it ideally suited for environments with complex layouts and multiple obstructions where traditional technologies often fail [5,6].

Traditional navigation technologies, such as Global Positioning System (GPS) and Light Detection and Ranging (LiDAR), although effective in many scenarios, exhibit significant limitations under certain conditions. GPS, for example, is susceptible to signal degradation in indoor or urban environments due to its reliance on satellite signals, which can be obstructed or reflected [7,8,9]. LiDAR, while offering high-resolution environmental mapping, is hindered by its high operational costs and reduced effectiveness in varying light conditions or in environments with airborne particulates, which are typical in many industrial settings [10,11,12].

In contrast, UWB technology offers substantial advantages in terms of both accuracy and reliability. It is known for its ability to maintain high levels of performance in environments with physical and radio frequency interference, which are common in industrial applications. The technology’s high data transmission rates and low power requirements further enhance its suitability for industrial applications where efficiency and power management are critical [13,14,15,16]. However, despite these advantages, UWB is not without its limitations:

1. Scalability: Expanding UWB systems to cover large industrial areas can be costly and complex. The need for numerous base stations and infrastructure can limit its scalability in extensive operations [17,18].
2. Initial Setup Cost: The initial cost of implementing UWB technology is high, which can be a barrier to widespread adoption, especially for smaller enterprises or those with limited budgets [19].
3. Multipath Interference: In environments with many reflective surfaces, UWB signals can experience multipath interference, which can affect the accuracy of location tracking [7,20].
4. Signal Attenuation: UWB signals can be attenuated by obstacles and materials commonly found in industrial settings, such as metal structures, which can impact performance [21,22].
5. Synchronization Requirements: Accurate synchronization between UWB devices is crucial for maintaining precision, and achieving this synchronization can be technically challenging [23,24].

Despite these challenges, UWB technology remains a promising solution for enhancing the positioning and navigation capabilities of AGVs in industrial environments. This research aims to thoroughly investigate the application of UWB technology for precise positioning and navigation of autonomous industrial trucks. By conducting a detailed comparative analysis using GPS and LiDAR within a controlled industrial environment, this study evaluates UWB’s effectiveness under a variety of operational conditions. Specifically, we address the challenges of positioning accuracy, reliability under diverse environmental conditions, and operational efficiency. The objective is to provide a robust empirical basis for recommending UWB’s broader adoption in industrial AGV applications [20,21,25,26].

Furthermore, this study addresses a notable gap in the existing literature, which often neglects the practical aspects of implementing advanced positioning technologies in real-world settings. Through empirical testing and systematic analysis, this work explores not only the technical feasibility but also the operational, economic, and safety implications of UWB technology in industrial settings [22,27,28]. We highlight the innovative use of UWB technology to overcome the limitations of existing systems, providing insights into its practical benefits and integration potential [23,24].

The methodology of this study involves comprehensive testing across multiple scenarios to assess the accuracy, reliability, and efficiency of UWB compared to traditional positioning technologies. This approach is underpinned by a solid theoretical foundation and draws on data collected from extensive field trials in industrial environments [29,30,31]. We also discuss the scalability and cost considerations, providing strategic recommendations for UWB’s implementation in industrial settings [32].

The expected outcomes of this research include a detailed assessment of UWB technology’s performance metrics, insights into its practical benefits, and strategic recommendations for its implementation. These findings are intended to contribute significantly to the field of AGV technology, supporting the advancement of UWB systems as a superior choice for industrial autonomous vehicle applications [33,34,35].

In conclusion, this introduction provides an extensive overview of the research context, elucidating the transformative potential of UWB technology in industrial automation. The following sections will delve deeper into the experimental design, and data analysis, and discuss the broader implications of the findings, aiming to provide a comprehensive resource for advancing the field of AGV technology in complex and dynamic industrial environments [36,37,38,39].

## 2. Traditional RTLS-BASED AGV Truck Navigation Systems

Real-Time Location Systems (RTLSs) greatly bolster the navigation capabilities of AGVs both indoors and outdoors. A substantial body of research supports the integration of RTLSs to refine AGV navigation, highlighted by numerous studies referenced in [3,4,6,9,11,12,15,16,18,19,20,21]. This section will offer an in-depth examination of the principal characteristics of RTLS-based safety protocols currently utilized in AGV systems.

### 2.1. Sensor Fusion of LiDAR, GPS, Image-Based Positioning, and UWB

Autonomous vehicles rely on a combination of technologies to navigate complex environments effectively. Each technology—LiDAR, GPS, image-based positioning, and Ultra-Wideband (UWB)—brings unique strengths to this multifaceted challenge.

**LiDAR** offers high-resolution distance measurements in a 3D format, essential for precise navigation and real-time obstacle detection. However, it can be expensive and performs poorly in adverse weather conditions such as fog or heavy rain, which scatter its laser beams.

**GPS** provides crucial geographical positioning over long distances but suffers from signal obstruction in urban canyons or densely covered natural environments, leading to significant positioning errors.

**Image-based positioning** leverages cameras to capture detailed visual information from the vehicle’s surroundings. Cameras are particularly useful for recognizing road signs, lane markings, and traffic signals, integrating visual context that LiDAR and GPS cannot provide. However, the main limitation of cameras is their inability to perceive depth accurately from a single image and their performance dependency on lighting conditions.

Integrating these technologies with UWB can significantly enhance the overall positioning system. UWB is known for its high accuracy and low latency in distance measurements, making it ideal for precise, close-range positioning tasks. Unlike GPS, it does not rely on external signals from satellites, which makes it highly reliable and secure. UWB’s ability to penetrate obstacles allows for effective operation even in environments where other signals might be obstructed.

The fusion of LiDAR, GPS, image-based positioning, and UWB:

The integration of these sensors can be approached through various fusion strategies:

**Early Fusion**: This method involves merging raw data from all sensors (LiDAR point clouds, GPS coordinates, camera images, and UWB distance measurements) at the input level. This data-rich approach can be processed to create a comprehensive and nuanced understanding of the environment.

**Middle Fusion**: Features or information extracted from each sensor are combined at an intermediate step. For instance, LiDAR data can be used to inform the depth of understanding of images captured by cameras, while GPS provides a geospatial context, and UWB ensures precision in the immediate vicinity.

**Late Fusion**: In this approach, data from each sensor are processed separately, and the results are combined at a decision-making level. This might involve using camera and LiDAR data for object detection and localization, GPS for route planning, and UWB for precise maneuvering in tightly controlled spaces.

By leveraging the complementary capabilities of LiDAR, GPS, image-based positioning, and UWB, autonomous vehicles can achieve a higher level of situational awareness and operational accuracy. This sensor fusion not only compensates for the individual weaknesses of each sensor type but also synergistically enhances their strengths, leading to more reliable and safer autonomous navigation systems.

In conclusion, the integration of these diverse sensing technologies through sophisticated fusion techniques represents the forefront of current research in autonomous vehicle navigation. The combined use of LiDAR, GPS, camera-based systems, and UWB forms a robust framework that significantly advances the reliability, safety, and efficiency of autonomous driving solutions.

### 2.2. Basic Architecture of a Real-Time Location System

**Figure 1:**
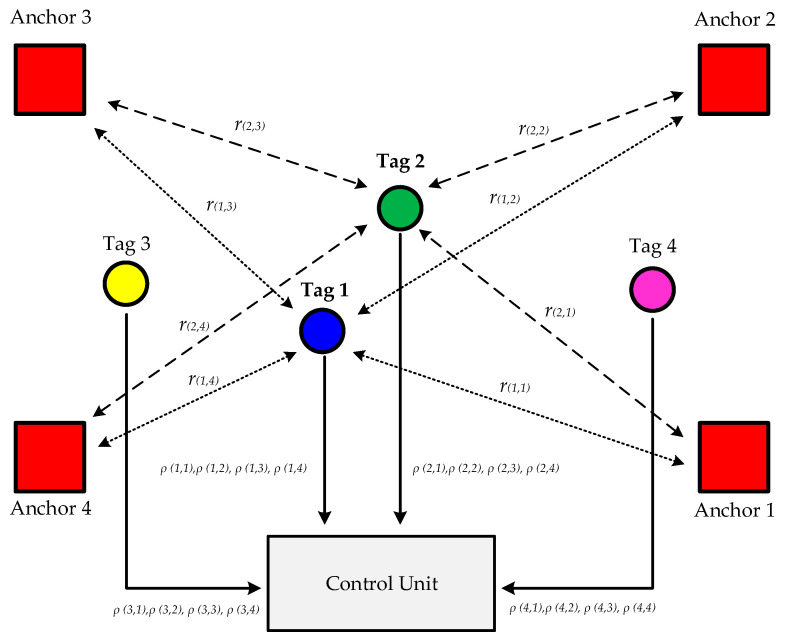
Schematic diagram of a Fixed-Infrastructure Real-Time Location System (FI-RTLS) designed for use in both indoor and outdoor environments [42].

A typical radio frequency (RF) location system for indoor and outdoor environments consists of a set of anchors with known positions (*xanj*, *yanj*, and *zanj*) and one or more tags whose positions (*xi*, *yi*, and *zi*) are to be determined [22,23,24,31,32,35,38,39,40,41,42]. These systems rely on a fixed infrastructure composed of anchors, referred to as Fixed-Infrastructure RTLSs (FI-RTLSs). Figure 1 shows the basic architecture, which includes multiple anchors, tags, and a Control Unit (CU). The location of each tag is estimated through two main steps: ranging and positioning.

Initially, the ranging measurements (angle, received power, or Time of Flight) between each anchor and the tags, denoted as ${\hat{\rho }}_{\left(i,j\right)}$, are determined using specific algorithms, as cited in [38,43,44,45,46,47,48]. These measurements often diverge from the actual values *ρ_(i_*_,*j*)_ due to potential transmission errors or multipath interference, which can degrade the signals. Subsequently, the position of each tag, represented by coordinates (*x_i_*, *y_i_*, and *z_i_*), is computed in the CU utilizing these ranging estimates. Figure 1 also illustrates the transmitted signal *r_(i_*_,*j*)_ from the *j^th^* anchor to the *i^th^* tag.

The system depicted in Figure 1 is known as a tag-based system. In this system, the tag is responsible for gathering the ranging measurements, noted as ${\hat{\rho }}_{\left(i,j\right)}$, and transmitting them to the CU. Additionally, the tag can calculate its own position based on these measurements and then forward these position estimates to the CU. Conversely, in an anchor-based system, it is the anchors that collect the ranging measurements and send them to the CU, where the position is then calculated.

One of the primary challenges faced by RF-based indoor and outdoor location systems is the multipath effect. Indoor and outdoor environments often produce multiple copies of the transmitted signal due to reflections from nearby objects. Sometimes, the direct path may be obscured and not represent the strongest signal path. This scenario typically results in significant degradation of ranging performance in narrowband location systems, consequently reducing location accuracy. A practical method to counteract the multipath effect is to expand the bandwidth of the signal transmitted by the tag [49]. Therefore, the extensive bandwidth of Impulse Radio Ultra-Wideband (IR-UWB) positions it as an ideal candidate for an RTLS intended for both indoor and outdoor use [50].

### 2.3. Navigation System for AGVs Using an FI-RTLS

In intralogistics environments, AGVs operate collectively within a fleet. The coordination of the fleet is managed by a Fleet Control System (FCS), which integrates production data from the factory’s Enterprise Resource Planning (ERP) system. With these data, the FCS issues directives to the AGVs specifying their starting points, destinations, and the routes they should follow. In the absence of a Real-Time Location System (RTLS) capable of identifying the locations of AGVs, other mobile robots, and personnel, routing decisions are made without considering the current positions of these entities. This oversight can cause the FCS to direct traffic through busy areas, leading to avoidable halts and a decrease in overall plant efficiency. Consequently, it is evident that the locational insights provided by an RTLS significantly enhance the navigational efficiency of AGV fleets.

**Figure 2:**
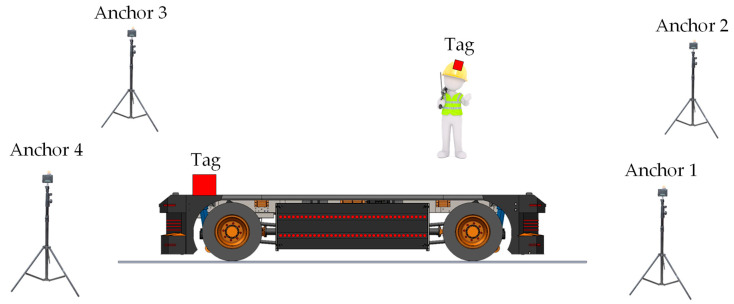
Core layout of a Fixed-Infrastructure Real-Time Location System (FI-RTLS) for AGV truck navigation in an industrial setting.

Figure 2 illustrates the essential layout of an FI-RTLS designed for AGV navigation. In this setup, anchors are strategically placed on the plant’s infrastructure, such as poles and walls, while both AGVs and personnel are equipped with tags. In a tag-based FI-RTLS configuration, the tag locations are wirelessly transmitted to the FCS, potentially using the same IR-UWB communication link as the position estimation or an alternative link. This process increases the tags’ power consumption, thereby shortening their battery life. In contrast, an anchor-based FI-RTLS has a Control Unit that gathers all ranging data, calculates the position estimates, and then forwards these estimates to the FCS.

Both scenarios require an ultra-low latency communication link between the FCS and the AGVs, especially if the FCS’s commands are intended to serve as a safety mechanism, such as stopping an AGV to avoid a collision. Although 5G technology is designed to provide the necessary low latency for such applications, its widespread implementation is still in progress. Until 5G becomes widely available, the positional data from the FI-RTLS AGV navigation system are mainly used by the FCS to monitor AGV movements.

A major drawback of current FI-RTLS AGV navigation systems is their dependence on anchors installed at various locations within the plant. The accuracy of these systems is highly influenced by the placement of the anchors, requiring meticulous planning during the implementation phase of an FI-RTLS. Many FI-RTLSs necessitate specific on-site calibration to achieve the desired accuracy [51]. Given the high product variability and short product life cycles prevalent in modern industries, production plant layouts frequently change, affecting the accuracy of an FI-RTLS. Any modification in the layout may require repositioning the anchors and/or repeating the calibration process, which can be both time-consuming and costly.

## 3. Overview of the Proposed FI-RTLS AGV System

This section can be divided into subheadings to provide a clear and detailed explanation of the proposed FI-RTLS AGV system. It will cover the design and implementation of the system, the results of experimental tests, the interpretation of these results, and the conclusions derived from the experiments.

### 3.1. Proposed Architecture

**Figure 3:**
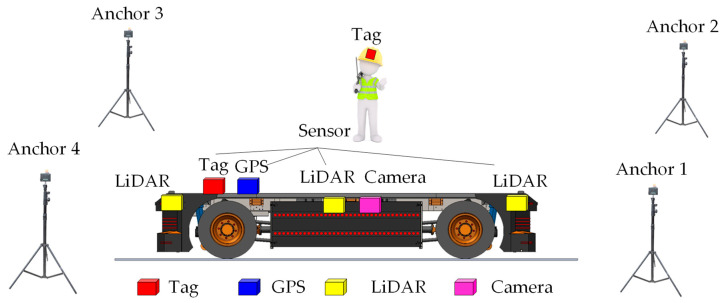
The architecture of the proposed hybrid FI-RTLS AGV truck safety system with multi-sensors.

Figure 3 presents the architecture of the proposed UWB system with LiDAR, GPS, and camera functionality, enhancing navigation and safety systems for AGVs. This setup differs from traditional RTLS-based solutions. The proposed AGV safety system should be installed only in crowded and narrow areas at risk of collision. The electric AGV truck is equipped with tags, LiDAR, GPS, and camera sensors, enabling it to determine the real-time location of moving people or assets. Therefore, the proposed safety system is based on real-time location within fixed infrastructure in specific crowded and narrow areas. In open areas or where there is sufficient road space, specialized navigation systems using LiDAR, GPS, and cameras will detect locations and serve as navigation aids.

This safety system is termed hybrid FI-RTLS AGV, as it incorporates both fixed and non-fixed infrastructure, combining the advantages of traditional FI-RTLS AGV systems. When operating in open areas, navigation through sensors installed on the vehicle suffices. There is no need to install numerous anchors throughout the factory area. However, in narrow spaces with many obstacles or in warehouse buildings, a UWB system will be employed to enhance positioning accuracy. The number of anchors installed depends on the plant layout. The hybrid FI-RTLS AGV system facilitates easy deployment of AGV safety and enhances usability in recycling plants. Additionally, the proposed system will enable the factory to adapt flexibly to the demands of the industry 4.0 manufacturing scenario.

**Figure 4:**
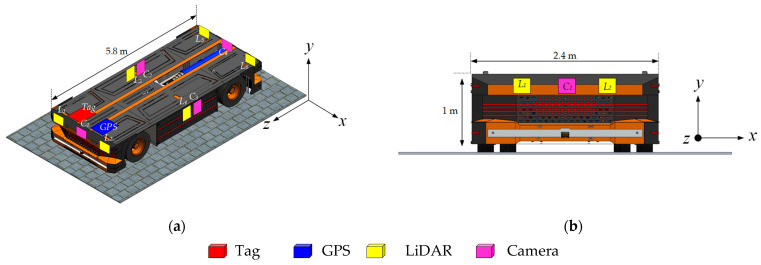
Placement of the tag, LiDAR, GPS, and camera sensors in the E-AGV truck: (**a**) perspective view; (**b**) front view.

Figure 4 shows the sensor configuration on an electric E-AGV truck. The arrangement comprises six LiDAR sensors strategically positioned at the vehicle’s corners and along both lateral sides. Additionally, four cameras are affixed at the front, rear, left, and right facades of the truck to facilitate comprehensive visual monitoring. Navigation and identification capabilities are augmented by a GPS unit and a tagging mechanism, both installed at the vehicle’s forefront. These sensors engage in communication via IR-UWB with mobile objects tagged within the operational environment, such as factory workers’ helmets or other mobile assets. This interaction utilizes the Time of Flight (ToF) data of the emitted signals to accurately estimate the distance between each tag and the sensors on the E-AGV truck, thus enabling precise object localization around the electric autonomous truck.

### 3.2. Implementation of UWB Positioning Using the TDoA Algorithm

In our prior research, we introduced an Ultra-Wideband (UWB) positioning system that utilized Two-Way Ranging (TWR) techniques and non-line-of-sight (NLOS) mitigation strategies [52]. However, we observed that the interval required for positioning expanded significantly with an increase in the number of devices to be located. To address this issue, this study implements the Time Difference of Arrival (TDoA) algorithm [53], which maintains a consistent positioning interval regardless of the number of devices.

**Figure 5:**
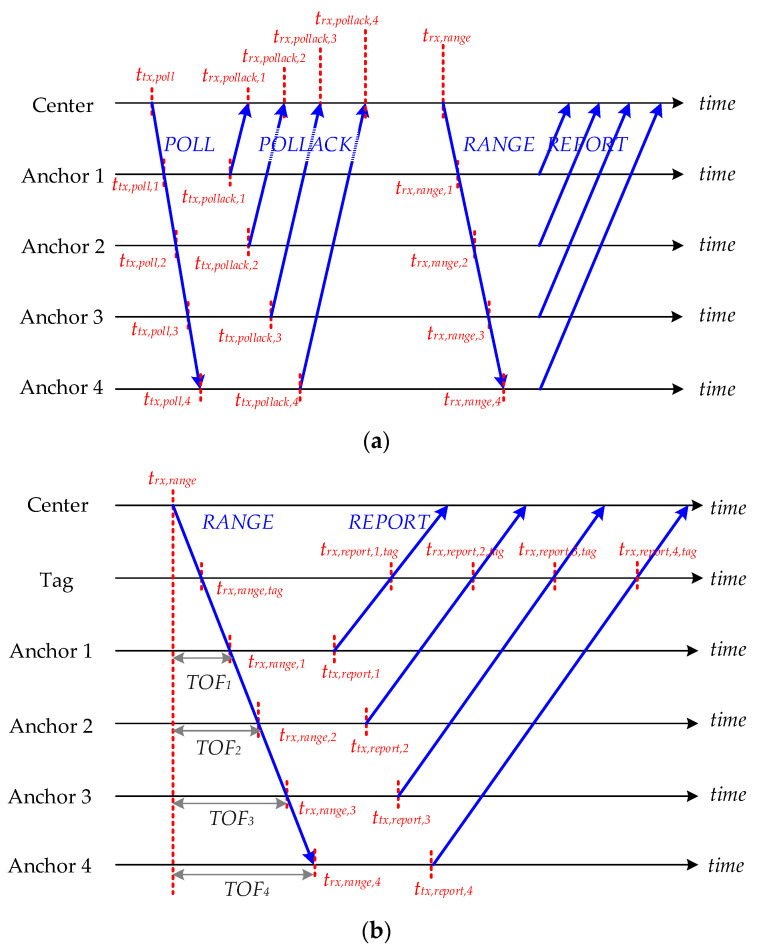
(**a**) The modified TWR between the center and anchors. (**b**) TDoA with anchor synchronization.

The successful deployment of the UWB system employing the TDoA technique hinges on the precise synchronization of anchors. This necessity arises because the clock frequency ratio (CFR) and the transmission time offsets differ across devices. The CFR, crucial for this setup, is determined by comparing the timestamps generated by two devices over the same period. For example, for *Anchor_i_* depicted in Figure 5a, the CFR is formulated as follows:

(1) $$r_{Anchor,i}=\frac{t_{rx,rang,i}-t_{rx,poll,i}}{t_{rx,ramge}-t_{rx,poll}}$$

The TOF between the central unit, which initiates the communication, and *Anchor_i_*, measured according to the central unit’s clock, can be expressed as follows:

(2) $$T_{i}=\frac{(t_{rx,pollack,i}-t_{tx,poll})-\frac{t_{tx,pollack,i}-t_{rx,poll,i}}{r_{Anchor,i}}}{2}$$

This modified TWR method is designed to calculate the distance using the central unit’s clock. As shown in Figure 5b, the tag captures all signals from both the central unit and the *Anchor_i_*. By receiving the poll and range signals from the central unit, the CFR of a tag can be expressed as follows:

(3) $$r_{tag}=\frac{t_{rx,range,tag}-t_{rx,poll,tag}}{t_{tx,range}-t_{tx,poll}}$$

Next, the synchronized timestamp for *Anchor_i_* is as follows:

(4) $${t^{\prime }}_{rx,report,i,tag}=t_{rx,report,i,tag}-r_{tag}\times T_{i}-(t_{tx,report,i}-t_{rx,range,i})\frac{r_{tag}}{r_{Anchor,i}}$$

**Figure 6:**
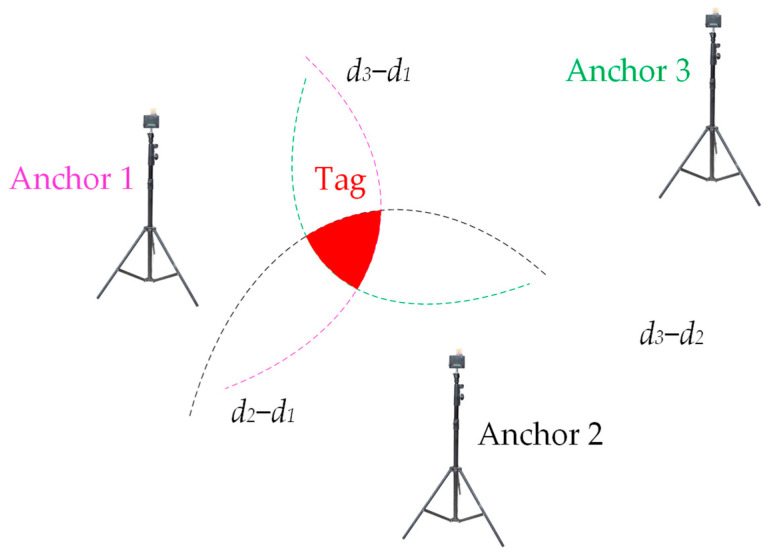
The hyperbolic curves may converge within an area rather than at a single point. The exact location of the tag must be inferred from this region.

These synchronized timestamps are instrumental in TDoA calculations when compared with the received timestamp from the central unit. In this method, the tags solely act as receivers, allowing for an unlimited number of tags. By using the synchronized time differences between the central unit and the anchors, the differences in distances can be determined by multiplying these time intervals by the speed of light in air. Nonetheless, the resulting hyperbolic curves derived from these distance differences may not converge to a single point but might overlap within a specific area, as illustrated in Figure 6. To accurately determine the location of a tag, the TDoA positioning algorithm is employed to estimate its position based on where these hyperbolas intersect.

Assume the coordinates of the *i_th_* anchor are [*x_i_*, *y_i_*], and the estimated coordinates of the tag are [*x*, *y*]. The measured distance difference between the *i_th_* anchor and the *j_tn_* anchor is *d_ij_*, while *d_i_* represents the distance between the estimated tag position and the *i_th_* anchor. The objective of the algorithm is to minimize the following loss function:

(5) $$f=\sum_{i>j}(\sqrt{{\left(x-x_{i}\right)}^{2}+{\left(y-y_{i}\right)}^{2}}-\sqrt{{\left(x-x_{j}\right)}^{2}+{\left(y-y_{j}\right)}^{2}}-{d_{ij}}^{2}$$

One common approach is the least-squares (LS) closed-form solution. The relationships among *d_i__j_*, *d_i_*, and [*x_i_*, *y_i_*] can be represented in matrix form as follows [53,54]:

(6) Aθ=b

where

(7) $$A=\left[\begin{array}{ccc} x_{2}-x_{1} & y_{2}-y_{1} & d_{21}\\ \begin{array}{l} x_{3}-x_{1} \end{array} & \begin{array}{l} y_{3}-y_{1}\\ \vdots \end{array} & \begin{array}{l} d_{31} \end{array}\\ x_{n}-x_{1} & y_{n}-y_{1} & d_{n1} \end{array}\right],\;\theta =\left[\begin{array}{c} x\\ y\\ d_{1} \end{array}\right],\;b=\left[\begin{array}{c} x_{2}^{2}+y_{2}^{2}-x_{1}^{2}-y_{1}^{2}-d_{21}\\ x_{3}^{2}+y_{3}^{2}-x_{1}^{2}-y_{1}^{2}-d_{31}\\ \vdots \\ x_{n}^{2}+y_{n}^{2}-x_{1}^{2}-y_{1}^{2}-d_{n1} \end{array}\right]$$

The solution can be formulated as follows:

(8) $$\theta =(A^{Τ}{A)}^{-1}A^{Τ}b$$

Another solution is the Chan method. The method is based on a twice LS solution, and it is widely used in TDoA estimation [54,55,56]. However, the estimated position is not precise enough by only using LS and the Chan method. The Taylor method is a recursive method with an initial position. The displacement in each iteration can be calculated using the following equation [54,56,57]:

(9) $$\delta_{Taylor}=\left[\frac{\Delta x}{\Delta y}\right]={\left(G^{T}Q^{-1}G\right)}^{-1}G^{T}Q^{-1}h$$

where

$$G=\left[\begin{array}{cc} (x_{1}-x)/d_{1}-(x_{2}-x)/d_{2} & (y_{1}-y)/d_{1}-(y_{2}-y)/d_{2}\\ (x_{1}-x)/d_{1}-(x_{3}-x)/d_{3} & (y_{1}-y)/d_{1}-(y_{3}-y)/d_{3}\\ \vdots & \vdots \\ (x_{1}-x)/d_{1}-(x_{n}-x)/d_{n} & (y_{1}-y)/d_{1}-(y_{n}-y)/d_{n} \end{array}\right],$$

$$h=\left[\begin{array}{c} d_{21}-(d_{2}-d_{1})\\ d_{31}-(d_{3}-d_{1})\\ \vdots \\ d_{n1}-(d_{n}-d_{1}) \end{array}\right],$$

$$Q=\left[\begin{array}{cccc} \mathrm{std}\;(d_{21}) & 0 & \cdots & 0\\ 0 & \mathrm{std}\;(d_{31}) & & 0\\ \vdots & & \ddots & 0\\ 0 & 0 & 0 & \mathrm{std}\;(d_{n1}) \end{array}\right].$$

By iteratively adjusting the estimated position of a tag until the displacement becomes sufficiently minimal, the precision of the tag’s location can be enhanced. However, in certain cases, the estimated position derived using the Taylor method deviates significantly from the actual position due to the small determinant of ***G*^T^*Q*^−1^*G***.

The Gradient Descent (GD) method is another iterative technique that starts with an initial position [58,59]. The adjustments to the position are informed by the gradients derived from the partial differentials of Equation (5), calculated as follows:

(10) $$f_{x}=2\sum_{i>j}[(\sqrt{{\left(x-x_{i}\right)}^{2}+{\left(y-y_{i}\right)}^{2}}-\sqrt{{\left(x-x_{j}\right)}^{2}+{\left(y-y_{j}\right)}^{2}}-d_{ij})\;\times (\frac{x-x_{i}}{\sqrt{{\left(x-x_{i}\right)}^{2}+{\left(y-y_{i}\right)}^{2}}}-\frac{x-x_{j}}{\sqrt{{\left(x-x_{j}\right)}^{2}+{\left(y-y_{j}\right)}^{2}}})]$$

(11) $$f_{y}=2\sum_{i>j}[(\sqrt{{\left(x-x_{i}\right)}^{2}+{\left(y-y_{i}\right)}^{2}}-\sqrt{{\left(x-x_{j}\right)}^{2}+{\left(y-y_{j}\right)}^{2}}-d_{ij})\;\times (\frac{y-y_{i}}{\sqrt{{\left(x-x_{i}\right)}^{2}+{\left(y-y_{i}\right)}^{2}}}-\frac{y-y_{j}}{\sqrt{{\left(x-x_{j}\right)}^{2}+{\left(y-y_{j}\right)}^{2}}})]$$

(12) $$\delta_{GD}=\left[\begin{array}{c} \Delta x\\ \Delta y \end{array}\right]=\left[\begin{array}{c} -f_{x}\\ -f_{y} \end{array}\right]$$

Rather than directly adjusting the coordinates [*x*, *y*] with the displacement, employing an adaptive gradient proves beneficial in locating the minimum of the loss function [60]. The Gradient Descent (GD) method enhances accuracy but requires more computational time compared to the Taylor method. A hybrid approach, termed the GD–Taylor method, is suggested by integrating these techniques. This approach considers both the gradient information and the Taylor series expansions, allowing for a refined adjustment of the displacement as follows:

(13) $$\delta_{GD-Taylor}=\delta_{GD}+\delta_{Taylor}$$

The detail of this method is outlined in Algorithm 1, starting with the computation of distance differences in Step 1, Steps 2 and 3 involve setting up the initial values for the adaptive gradient’s weight and the tag’s position. The core process, running from Step 4 to Step 15, iteratively adjusts the estimated position of the tag. In this main loop, Step 5 computes the distances from the anchor positions to the current estimate of the tag’s position. Following this, Step 6 generates the values for $\delta_{Taylor}$ and $\delta_{GD}$ based on Equations (9) and (12). Steps 7 and 8 adjust the $\delta_{GD-Taylor}$ values and update the *weights*, respectively. Steps 9 and 10 implement the modified adaptive gradient method, and Step 11 increments the iteration count. Steps 12 through 14 monitor the norm of the displacement to potentially halt the main loop prematurely if the displacement is sufficiently small. Ultimately, the refined estimated position of the tag is finalized and output in Step 16.

In the GD–Taylor method, the initial displacement is large to facilitate rapid convergence, primarily due to the influence of the Taylor method. As the iterations progress, the estimated position gradually approximates the actual position, and the displacement reduces sufficiently to meet the criteria for early termination in Step 12. Moreover, Steps 8 through 10 are crucial for regulating the displacement to prevent data overshoot that can occur with the Taylor method. Consequently, the GD–Taylor method effectively combines the strengths of both the Taylor and Gradient Descent methods, optimizing both calculation speed and positional accuracy.
**Algorithm 1. Function of the GD–Taylor method**.**Input**Locations of anchors (*x*_1_, *y*_1_), (*x*_2_, *y*_2_),…, (*x*_n_, *y*_n_) Received time stamps *t*_1_, *t*_2_,…, *t*_n_
Maximal iteration time *max_iter*
Initial location (*x*_init_, *y*_init_)**Output**Estimated location of tag (*x*_t_, *y*_t_)**1**Calculate *d*_21_, *d*_31_, …, *d*_n1_ by multiplying light speed and time resolution to (*t*_2_ *t*_1_), (*t*_3_ *t*_1_), …, (*t*_n_ *t*_1_);**2**Set weight to 10^−10^;**3**Set (*x*, *y*) to (*x*i_nit_, *y*_init_);**4****while** times < *max_iter* **do****5***d*_1_, *d*_2_, …, *d*_n_ are the distances from anchors to (*x*, *y*);**6**use (8) and (11) to calculate $\delta_{Taylor}$ and $\delta_{GD}$;**7**Set $\delta_{GD-Taylor}$ to ($\delta_{Taylor}$+$\delta_{GD}$);**8**Set weight to (*weight*+ $\delta_{GD-Taylor,\;x^{2}}$ + $\delta_{GD-Taylor,\;y^{2}}$);**9**Set *x* to (*x*+ $\delta_{GD-Taylor,\;x}$/(*weight*)^1/2^);**10**Set *y* to (*y*+ $\delta_{GD-Taylor,\;y}$/(*weight*)^1/2^);**11***times*++;**12****if** (($\delta_{GD-Taylor}$, *x*^2^+ $\delta_{GD-Taylor}$, *y*^2^)/*weight*)^1/2^ < 0.001 **then****13****break****14****end if****15****end while****16****return** (*x*, *y*)

For comparison, the methods presented in [53] are similar to the GD–Taylor method for position calculations. The GD–Taylor method is chosen for its ability to handle the non-linear, noisy, and dynamic nature of industrial environments, providing a robust, accurate, and adaptable solution for enhancing autonomous truck navigation with UWB technology. This ensures that the autonomous trucks can operate safely and efficiently, meeting the demands of modern industrial operations.

### 3.3. Extend Kalman Filter (EKF) Node

In this section, we delve into the ‘*ekf_localization_node’*, a specialized Kalman Filter (KF) implementation, designed specifically for the real-time state estimation of AGVs within the ‘*robot_localization*’ package. This package forms part of a comprehensive framework developed for the Robot Operating System (ROS), aimed at facilitating advanced navigation and positioning capabilities in AGV systems.

The ‘*ekf_localization_node’* serves as a critical component of our localization architecture, executing complex algorithms to fuse data from diverse sensor inputs. This node is engineered to continuously estimate the AGV’s position and orientation by integrating measurements from various onboard sensors. These may include, but are not limited to, Light Detection and Ranging (LiDAR), GPS receivers, cameras, UWB sensors, and wheel encoders, each providing vital data necessary for accurate localization.

Key features of the ‘*ekf_localization_node*’ include the following:

**Multi-Sensor Fusion**: The node is capable of processing an unlimited number of inputs from different sensor types. This capability is crucial for AGVs operating in dynamic environments where multiple data streams must be synthesized to form an accurate estimation of the vehicle’s state.

**Customizability**: Users can specify which sensor data fields should be integrated into the state estimation process. This flexibility allows the system to adapt to various sensor configurations and ensures that the node can be tailored to meet the specific needs of any AGV application.

**Robust Algorithm Implementation**: At the core of the ‘*ekf_localization_node*’ is the Extended Kalman Filter algorithm, renowned for its effectiveness in dealing with non-linear systems typical of robot navigation. The EKF approximates the state of a dynamic system using a series of measurements observed over time, which are subject to noise and other inaccuracies.

**Real-Time Performance**: Designed to operate in real time, the node ensures minimal latency in processing and updating the vehicle’s state. This is imperative for maintaining the operational efficiency and safety of AGVs, particularly when navigating through unpredictable or complex environments.

The ‘*ekf_localization_node*’ not only enhances the positional accuracy and operational reliability of AGVs but also serves as a scalable solution adaptable to a wide range of industrial applications. Its integration within the ‘*robot_localization’* package highlights our commitment to developing versatile, robust solutions for autonomous vehicle navigation, supporting the broader objectives of automation and efficiency in industrial logistics and manufacturing processes.

The EKF is a crucial tool in robotic navigation [61,62,63], helping to estimate the full 3D pose (position and orientation) and velocity of a mobile robot over time. This process treats the robot’s motion as a non-linear dynamic system, described by the following equation:

(14) $$x_{k}=f(x_{k-1})+w_{k-1}$$

where $x_{k}$ represents the robot’s state vector, including its 3D pose and velocity at time *k*, *f* denotes a non-linear state transition function, and *w*_*k*−1_ is the process noise, assumed to be normally distributed.

The state vector, *x*, encompasses the vehicle’s 3D pose and orientation, along with their respective velocities, with rotational values expressed using Euler angles. The system receives sensor measurements modeled as follows:

(15) $$z_{k}=h(x_{k})+v_{k}$$

where *z*_*k*_ is the measurement vector at time *k*, *h* is a non-linear sensor model mapping the state into measurement space, and *v*_*k*_ is the measurement process noise vector, normally distributed.

In the initial phase of the algorithm, illustrated by Equations (16) and (17), we execute a prediction step that extends the current state estimation forward in time:

(16) $${\hat{x}}_{k}=f(x_{k-1})$$

(17) $${\hat{P}}_{k}=FP_{k-1}F^{T}+Q$$

In this context, *f* represents a conventional 3D kinematic model based on Newtonian principles. The predicted error covariance, *P*, is extrapolated using state transition matrix (*F)* and the Jacobian matrix of *f*, and is subsequently adjusted by *Q*, the covariance of the process noise.

Subsequently, the correction phase is handled through Equations (18)–(20):

(18) $$K={\hat{P}}_{k}H^{T}{\left(H{\hat{P}}_{k}H^{T}+R\right)}^{-1}$$

(19) $$x_{k}={\hat{x}}_{k}+K(z-H{\hat{x}}_{k})$$

(20) $$P_{k}=(I-KH){\hat{P}}_{k}{\left(I-KH\right)}^{T}+KRK^{T}$$

The Kalman gain, *K*, is derived using the observation matrix *H*, the measurement covariance *R*, and the predicted error covariance ${\hat{P}}_{k}$. This gain is utilized to refine both the state vector and its covariance matrix. We implement the Joseph form update for the covariance to enhance the filter’s stability, ensuring that the covariance matrix remains positive semi-definite.

In standard EKF processes, *H* is expected to be the Jacobian matrix of the measurement function *h*. Given our system’s compatibility with a wide range of sensors, we operate under the assumption that each sensor contributes data relevant to the state variables being estimated. Hence, *H* is often configured as the identity matrix, simplifying integration and analysis. The *ekf_localization_node* accommodates partial state updates, which are crucial for dealing with sensor data that do not measure all state variables—this is typically the norm. In practical terms, when only a subset of *m* state variables is measured, *H* is reshaped into an *m* by 12 matrixes of range *m*, where non-zero values correspond directly to the columns of measured variables.

Furthermore, due to the challenges of precisely tuning the process noise covariance (*Q*), the *ekf_localization_node* offers this matrix as an adjustable parameter, allowing users to modify it according to the specific needs of their application [64]. This adaptability is crucial for fine-tuning the filter’s performance across different operational contexts.

In our implementation of the Extended Kalman Filter (EKF) for sensor fusion, we undertake several critical steps to ensure the accurate provision of statistical features of sensor output signals. These steps enhance the robustness and reliability of our state estimation.

**Noise Characteristics Determination**:

- Empirical Analysis: For each sensor (LiDAR, GPS, camera, UWB), we collect extensive data under controlled conditions. This involves multiple testing scenarios to capture various operational states.
- Covariance Estimation: We estimate the noise covariance matrices (*Q* for process noise and *R* for measurement noise) based on the collected data. These matrices reflect the statistical properties of the sensor noise.

**Rigorous Sensor Calibration**:

- Bias and Variance Identification: Calibration procedures are performed to identify and correct systematic biases and measure the variance in sensor outputs. This includes static and dynamic calibration techniques to ensure the sensors provide accurate readings.
- Dynamic Calibration: We conduct continuous monitoring and recalibration during operation to account for environmental changes and sensor aging.

**Data Fusion Framework**:

- ROS *robot_localization* Package: We employ the ROS *robot_localization* package, which integrates data from multiple sensors using EKF. This package supports the specification of noise parameters for each sensor, allowing the EKF to effectively manage the statistical properties of the sensor data.
- Parameter Specification: We use accurate specification of sensor noise parameters in the configuration files, ensuring that the EKF can adapt to the different noise characteristics of each sensor type.

**Consistency Checks and Validation:**

- Cross-Validation with Ground Truth Data: We perform cross-validation by comparing the EKF outputs with ground truth data obtained from high-precision reference systems. This helps to validate the accuracy of the EKF implementation.
- Dynamic Adjustment: Based on validation results, we dynamically adjust the noise characteristics in the EKF to maintain optimal performance. This iterative process helps to refine the state estimates continuously.

By following this methodology, we ensure that the statistical features of the sensor outputs are accurately provided and effectively utilized within the EKF framework. This leads to reliable and robust state estimation, crucial for the accurate navigation and operation of autonomous systems in complex environments.

Therefore, the Extended Kalman Filter is crucial for our autonomous navigation system. Autonomous vehicles often encounter non-linear motion dynamics, such as turns and accelerations, which the EKF efficiently handles to provide accurate state estimation. Additionally, our system relies on multiple sensors, including LiDAR, GPS, cameras, and UWB, all of which have non-linear measurement models. The EKF adeptly manages these complexities, ensuring reliable fusion results. Furthermore, even in stable environments, variations such as changing terrain, obstacles, and sensor noise can impact navigation accuracy. The robustness of the EKF ensures that our system remains reliable under these conditions, enabling our autonomous trucks to navigate safely and efficiently.

## 4. Results and Discussion

In this section, we conduct an analytical evaluation of the accuracy of a newly proposed Fixed Infrastructure Real-Time Location System (FI-RTLS) equipped with multiple sensors, including LiDAR, GPS, and cameras, which is designed to enhance vehicle safety and facilitate automated guided vehicle (AGV) navigation in expansive open areas. The assessment is executed through a series of measurements under two distinct conditions.

Initially, a static measurement setup was established to emulate the dimensions of the AGV. The primary objective of this setup was to investigate the impact of the proposed post-processing algorithm on the system’s accuracy. Following these preliminary measurements, the sensors were installed on an actual custom-built AGV truck. Subsequently, both static and dynamic measurements were conducted to assess the performance of our proposed system within the operational environment of the recycling plant at Millcon Burapha Co., Ltd., Rayong, Thailand.

Through these dual scenarios, we aim to provide a comprehensive evaluation of the FI-RTLS’s accuracy in static conditions—where the AGV remains stationary—and dynamic conditions that mimic the real-world movements of an AGV within an industrial setting. This methodological approach is designed to ensure a thorough understanding of the system’s capabilities and limitations, thereby laying a foundational basis for its potential implementation across various industrial applications.

### 4.1. UWB System Configuration

Table 1 delineates the primary configuration parameters employed in these measurements. The sensors and tags utilized in the proposed security system are equipped with the DW1000 chip from Deca wave, adhering to the IEEE 802.15.4 standard [65]. The Ultra-Wideband (UWB) distance measurements between the anchor and the tag are ascertained using the Two-Way Ranging (TWR) method, as elaborated in [65]. These measurements are subsequently refined through a post-processing algorithm, details of which are exhaustively discussed in Section 3.2 and succinctly summarized in Table 1.

For intra-system communication within the proposed Fixed Infrastructure Real-Time Location System (FI-RTLS) designed for the safety of E-AGV trucks, various protocols are implemented to manage the simultaneous navigation of multiple persons and AGVs within the same space. According to the findings of this research, the TWR-Time Division Multiple Access (TDMA) protocol was found to be adequate for monitoring up to 20 individuals around an E-AGV truck. For scenarios necessitating a higher number of AGVs and tags, the Time Difference of Arrival (TDoA)-TDMA is recommended as the most efficient protocol, as endorsed in [66].

A notable limitation of TDoA-based FI-RTLSs is the prerequisite for synchronizing the clocks of all anchors, often necessitating wired connections among them. This requirement tends to complicate the installation process in industrial environments. In response, this study advocates a hybrid approach that integrates an FI-RTLS utilizing TDoA in narrowly confined and diverse environments while employing a multi-sensor navigation strategy involving LiDAR, GPS, and cameras in more expansive areas. This method effectively mitigates some of the installation challenges associated with traditional FI-RTLSs by obviating the need for wired connections among anchors over large areas, thereby simplifying the deployment process in industrial settings.

In this section, we elaborate on the processing methodology of UWB signals. The UWB signal processing involves the following steps:

(1) Signal Acquisition: UWB signals are acquired using specialized UWB transceivers (DW1000). These transceivers capture the raw data, which include Time of Flight (TOF) and received signal strength (RSS) data [21,35].
(2) Preprocessing: The raw data undergo preprocessing to remove noise and outliers. This is achieved using filtering techniques such as band-pass filtering to eliminate out-of-band frequency noise components [39].
(3) Range Estimation: The preprocessed signals are used to estimate the distance between the UWB transmitter and the tag receiver. This involves calculating the TOF and applying it to determine the range, given the speed of light [45].
(4) Positioning Algorithm: The estimated ranges from multiple anchors are fed into a positioning algorithm. We use trilateration to compute the precise position of the tag in the 2D/3D space [46,47].
(5) Error Correction: To enhance accuracy, error correction algorithms like the Kalman Filter are applied to smooth out the position estimates and reduce the impact of multipath effects and other inaccuracies. These steps ensure accurate and reliable UWB signal processing, leading to precise position estimation in industrial environments [48,50].

**Figure 7:**
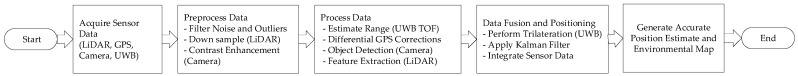
Flowchart of the signal processing workflow for integrating data from LiDAR, GPS, camera, and UWB sensors in an autonomous truck navigation system.

Figure 7 shows the process for integrating data from LiDAR, GPS, camera, and UWB sensors in an autonomous truck navigation system. The process begins with acquiring sensor data: LiDAR captures a 3D point cloud, GPS provides global positioning data, cameras capture visual images, and UWB measures Time of Flight and received signal strength to estimate distances. These raw data are then preprocessed: noise and outliers are filtered out, LiDAR data are down-sampled to reduce computational load, and camera images are enhanced through contrast adjustment.

Next, the preprocessed data are further processed according to each sensor’s specific requirements. UWB data are used to estimate distances by calculating the Time of Flight. GPS data are corrected using differential GPS techniques to improve accuracy. Camera images are analyzed to detect and classify objects using convolutional neural networks, and key features such as edges and surfaces are extracted from the LiDAR point cloud for mapping and obstacle detection.

The processed data from all sensors are then integrated to form a comprehensive understanding of the environment. Trilateration is performed on the UWB data to compute precise positions, and an EKF is applied to combine and smooth the data from different sensors, enhancing accuracy and reducing noise. The fused data are used to generate an accurate position estimate and create a detailed environmental map, which is essential for navigation and obstacle avoidance in the industrial environment.

This method ensures precise and reliable navigation for autonomous trucks, leveraging the strengths of multiple sensors and advanced data processing techniques to operate effectively in complex industrial settings.

### 4.2. First Measurement Campaign: Static Measurements with an E-AGV Truck Prototype

**Figure 8:**
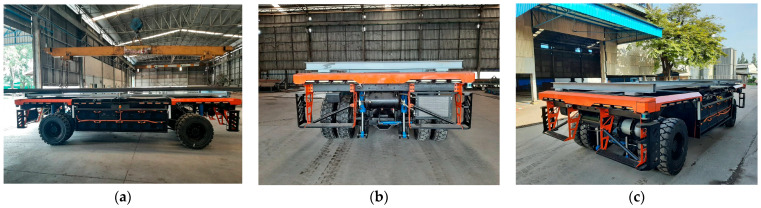
Prototype electric-automated guided vehicle (E-AGV) truck: (**a**) side view; (**b**) front view; and (**c**) perspective view.

Figure 8 shows a prototype electric Automated Guided Vehicle (AGV) truck engineered for enhanced navigational and safety features in industrial settings. This innovative model is designed to demonstrate advanced positioning capabilities using a combination of Ultra-Wideband (UWB), LiDAR, GPS, and camera technologies. The images show the truck from multiple angles, highlighting its robust structure and the integration of various sensors optimized for precise and safe autonomous navigation. This AGV prototype represents a critical step forward in developing reliable automated transport solutions for complex industrial environments.

**Figure 9:**
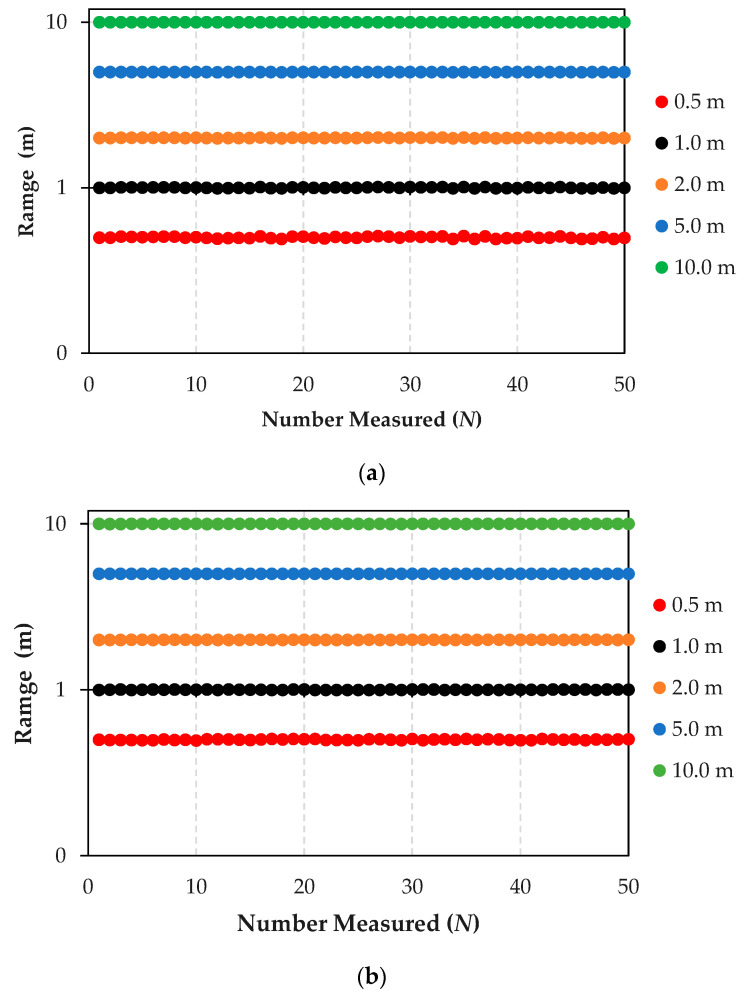
Measured results of advanced sensor integration for enhanced E-AGV truck navigation: (**a**) distance test using LiDAR, GPS, and camera sensors; (**b**) distance test using LiDAR, GPS, camera, and UWB sensors.

Figure 9 shows the results from rigorous distance testing aimed at assessing the navigational capabilities of E-AGV trucks, which are equipped with an array of sensors.

Figure 9a Distance Test Using LiDAR, GPS, and Camera Sensors: This part of the figure displays the outcome of a navigational test on an E-AGV truck, employing front-mounted sensors—LiDAR, GPS, and cameras—with an anchor serving as the transmission station. The test was conducted at varying distances—0.5, 1, 2, 5, and 10 m—with each distance point tested 50 times (*N*) to statistically analyze the results. The graph shows data processed using the Kalman Filter algorithm, demonstrating high accuracy and performance of the sensors with a minimal deviation of 0.6 cm from the test distances, translating to a percentage error of 0.084%. This high level of precision illustrates that these sensors are well suited for navigation in open, controlled environments like factory areas. However, in environments with limited space and poor visibility, the combination of LiDAR, GPS, and camera sensors might not suffice.

Figure 9b Distance Test Using LiDAR, GPS, Camera, and UWB sensors: This panel presents results from integrating an Ultra-Wideband (UWB) sensor with the existing setup of LiDAR, GPS, and camera sensors. The addition of UWB aims to enhance distance measurement accuracy, which is particularly useful in challenging environments where GPS signals are weak or obstructed, such as indoor settings, rainy conditions, or areas with high solar interference. The resulting data show a significant improvement in localization and positioning accuracy, with a deviation of only 0.235 cm from actual distances, corresponding to an error percentage of just 0.000045%. This remarkable accuracy proves especially beneficial for navigation in complex or dynamically changing industrial scenarios.

The comparative results from these tests underscore the enhanced accuracy in distance detection achieved through multi-layer sensor integration. This comprehensive analysis provides invaluable insights into the collective impact of these advanced sensor technologies on the operational efficiency of autonomous vehicles in various industrial settings.

**Figure 10:**
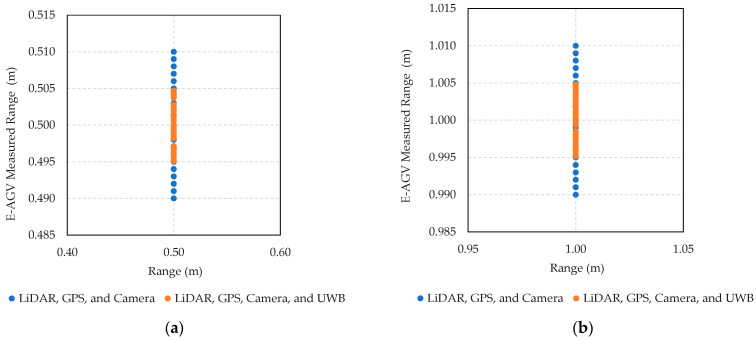

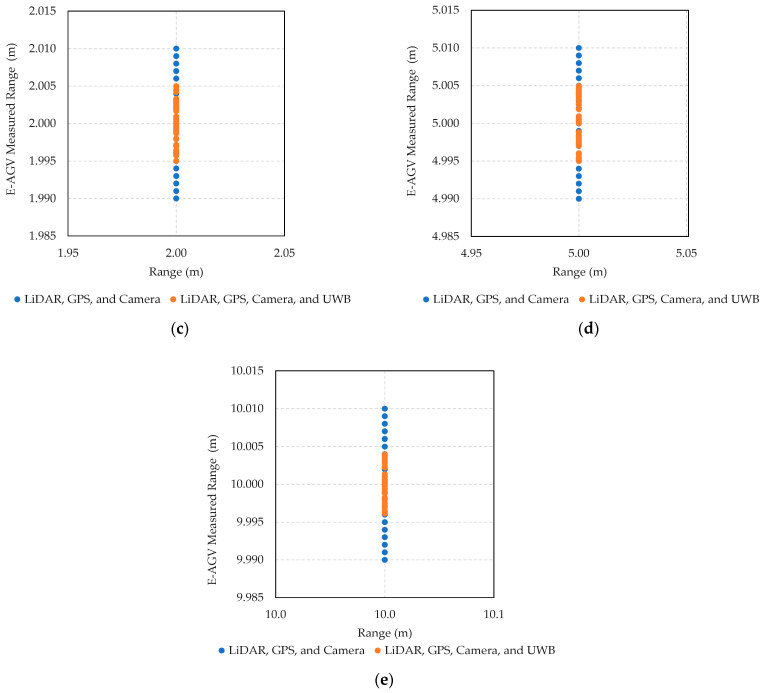
The results from distance measurement tests comparing the efficacy of LiDAR, GPS, and camera sensors to a configuration that includes an additional UWB sensor. The tests were repeated 50 times (*N*) at varying distances: (**a**) 0.5 m, (**b**) 1.0 m, (**c**) 2.0 m, (**d**) 5.0 m, and (**e**) 10.0 m.

Figure 10 presents the results from distance measurement tests comparing the efficacy of two case sensor configurations on an E-AGV truck. The first configuration includes LiDAR, GPS, and camera sensors, while the second additionally integrates an Ultra-Wideband (UWB) sensor. These tests were conducted at varying distances (0.5 m, 1.0 m, 2.0 m, 5.0 m, and 10.0 m) and each measurement was repeated 50 times to ensure statistical reliability.

At the 0.5 m distance (Figure 10a), the LiDAR, GPS, and camera setup exhibited measurements ranging from 0.490 to 0.510 m (blue dot), resulting in a maximum error of 2%. With the addition of the UWB sensor, the measured values were more tightly grouped from 0.495 to 0.505 m (orange dot), reducing the maximum error to 1%. This pattern of improved accuracy with the inclusion of UWB was consistent across all tested distances (Figure 10b–e). The integration of UWB enhances the precision of the navigation system, effectively reducing measurement errors, which is crucial for operational safety and efficiency in industrial settings. Additionally, the use of the Kalman Filter algorithm helps in correcting any discrepancies, further stabilizing and refining the system’s accuracy across various environmental conditions and operational scenarios.

### 4.3. Second Measurement Campaign: Static Measurements with an E-AGV Truck Prototype

**Figure 11:**
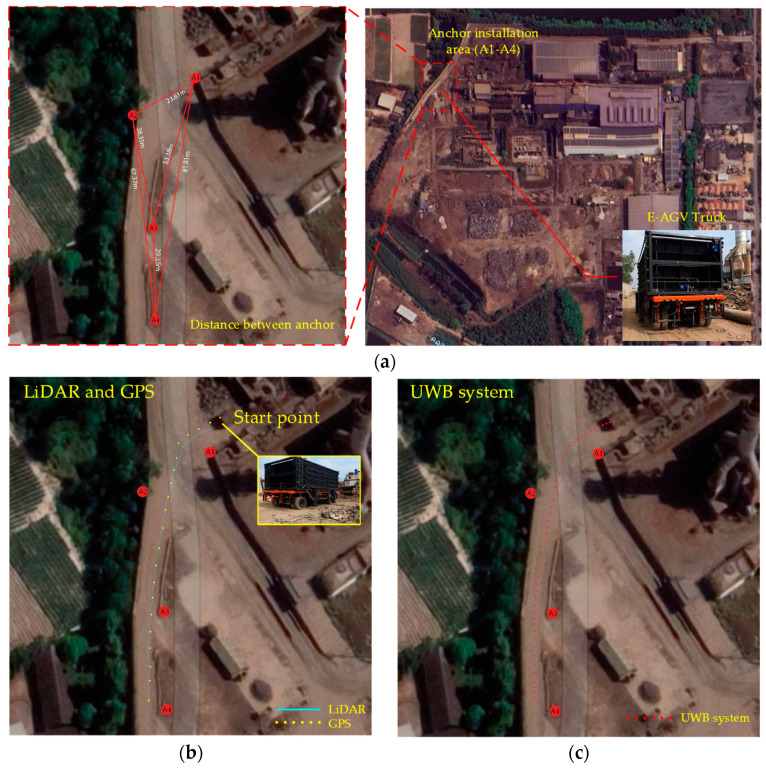

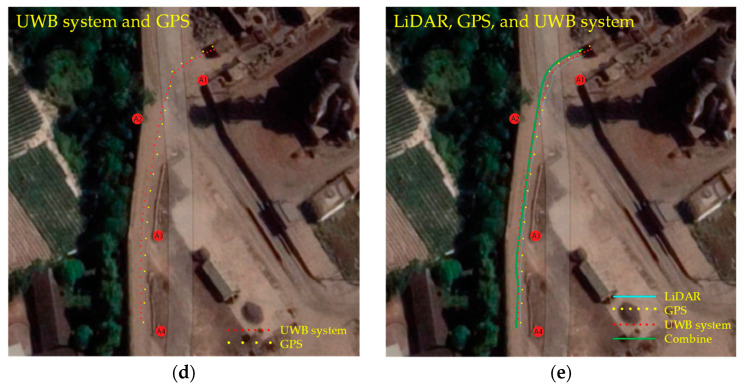
Testing autonomous electric trucks in real factory locations in conditions using different sensors for navigation: (**a**) installing anchors (A1–A4) in narrow road areas in factories; (**b**) GPS and LiDAR; (**c**) UWB sensor; (**d**) GPS and UWB sensors; and (**e**) LiDAR, GPS, and UWB sensors.

Figure 11 illustrates a comprehensive series of empirical tests conducted on an Electric Automated Guided Vehicle (E-AGV) within an actual factory environment, aimed at evaluating various sensor configurations for enhanced navigation capabilities. The sequence of images captures the systematic steps and settings throughout the testing process:

Figure 11a Installation of Anchors (A1–A4) in a Marrow Factory Roadway: This panel displays the preliminary setup phase, where anchors are strategically positioned along narrow factory corridors. These anchors serve as pivotal reference points for the Real-Time Location System (RTLS) and are essential for the precise functioning of the navigation system. The electric truck commences its route at the recycling material collection point, navigating through the constrained pathway and recording positional data as depicted in subsequent panels.

Figure 11b Integration of GPS and LiDAR: This image demonstrates the truck equipped with GPS and LiDAR sensors, navigating close to the right edge of the pathway yet maintaining its course towards the destination. The synergistic use of GPS and LiDAR not only enhances geolocation accuracy but also improves distance measurement, which is crucial for maneuvering through intricate routes and circumventing potential obstacles.

Figure 11c Deployment of UWB Sensor: Focusing on the implementation of Ultra-Wideband (UWB) sensors, known for their precision and minimal latency in distance measurements, the results indicate that the truck maintains a central path along the road, ensuring adequate clearance from roadside hazards, thereby highlighting the UWB’s efficacy in densely populated or cluttered factory settings.

Figure 11d Combination of GPS and UWB Technologies: This configuration illustrates how the amalgamation of GPS and UWB can mitigate the inherent limitations of each technology. While GPS provides extensive locational data albeit with less precision, UWB excels in delivering high accuracy over shorter ranges, thus ensuring robust navigational performance.

Figure 11e Comprehensive Sensor Array—LiDAR, GPS, and UWB: Displaying the full integration of LiDAR, GPS, and UWB, this setup aims to maximize the vehicle’s sensory input, from broad navigational support to meticulous obstacle detection and avoidance, enabling the AGV to make precise operational decisions as evidenced by the green trajectory line of the truck’s route.

Each image in Figure 11 underscores the practical advantages and the integration of multiple navigational technologies, optimizing both the operational efficacy and safety of autonomous vehicles in industrial contexts. The tests confirm the applicability of these technologies in real conditions, showcasing their potential to transform logistics and transportation in contemporary industrial landscapes.

## 5. Conclusions

This study investigates the application of UWB technology to enhance the positioning and navigation capabilities of autonomous industrial trucks, contrasting its performance with conventional systems such as GPS and LiDAR. The empirical findings underscore the principal advantages of UWB technology, notably its superior accuracy and robustness against environmental interferences that are prevalent in complex industrial settings. The experimental results reveal that UWB technology, when integrated with GPS and LiDAR, consistently achieves a positioning accuracy within 0.2 cm 99% of the time. This marks a substantial improvement compared to the accuracies of 10 cm and 5 cm typically observed with GPS and LiDAR systems, respectively. Even when GPS and LiDAR are used in conjunction, they only attain a navigation accuracy of about 2 cm. Additionally, UWB technology maintains high-performance levels even in challenging environments characterized by high metallic interference and non-line-of-sight conditions—scenarios where GPS and LiDAR effectiveness decrease by 40% and 25%, respectively. UWB’s performance remains stable and effective even under adverse environmental conditions such as rain, fog, or snow, showcasing its low power consumption and high efficiency in multi-user scenarios without signal interference.

The integration of UWB into existing logistical operations has been demonstrated to be feasible without necessitating substantial infrastructural modifications, further advocating for its adoption in industrial contexts. Nonetheless, this study also highlights potential challenges associated with UWB technology, including scalability, the costs related to initial setup, and the complexities of system integration. Although UWB technology has proven to be effective, the initial setup cost may be high, and there may be challenges in scalability. Further research is needed to explore how to reduce costs and improve scalability for wider adoption in industrial environments.

In conclusion, the findings from this study validate the hypothesis that UWB technology can significantly improve the operational capabilities of autonomous industrial trucks, offering a reliable and efficient resolution to the limitations inherent in current positioning technologies. It is recommended that industry stakeholders consider UWB as a strategic investment to advance autonomous operations in complex environments, potentially realizing significant advancements in precision and reliability for industrial logistics and transportation systems.

Future work will focus on reducing the cost of UWB systems through hardware design advancements and improving scalability by optimizing network architectures. Additionally, the research will explore robust integration protocols with existing technologies and enhance algorithm efficiency for better accuracy.

## Figures and Tables

**Table 1:** Configuration parameters of the UWB system.

Parameter	Value
Carrier frequency	3.9936 GHz
Bandwidth	500 MHz
Channel	5
Bitrate	6.8 Mbps
PRF (pulse repetition frequency)	16 MHz
Preamble length	1024 symbols
Preamble code	3
SFD (start of frame delimiter)	8 symbols
Latency	200 ms
Positioning rate	5 Hz
Tx power	−14 dBm

## Data Availability

Data are contained within the article.

## References

[1] Smith J.A., Brown B.C. (2021). Ultra-Wideband Positioning Systems for Industrial Environments. Ind. J. Nav. Tech..

[2] Johnson L., Lee M.K. (2020). Challenges of GPS Navigation in Urban and Indoor Settings. J. Geo. Res..

[3] Shi D., Mi H., Collins E.G., Wu J. (2020). An indoor low-cost and high-accuracy localization approach for AGVs. IEEE Access.

[4] Jiang J., Guo Y., Liao W. (2015). Research on AGV guided by real-time locating system (RTLS) for material distribution. Int. J. Control Autom..

[5] Thompson H., Walters T.Y. (2019). An Overview of LiDAR Technology and Its Automotive Applications. Automot. Innov. Rev..

[6] Kirsch C., Röhrig C. (2011). Global localization and position tracking of an automated guided vehicle. IFAC Proc..

[7] Wang X., Zhang Y. (2018). Improving Accuracy in Positioning Systems Using Ultra-Wideband Technology. Sens. Tech. J..

[8] White P.R., Green D.F. (2022). Integration Challenges of UWB in Autonomous Vehicles. J. Auto. Eng..

[9] Kirsch C., Künemund F., He D., Röhrig C. Comparison of localization algorithms for AGVs in industrial environments. Proceedings of the 7th German Conference on Robotics (ROBOTIK).

[10] Davis K., Murphy S. (2019). UWB vs. Traditional Positioning Technologies in Industrial Autonomous Vehicles. Ind. Auto. J..

[11] Pérez-Rubio M.C., Losada-Gutiérrez C., Espinosa F., Macias-Guarasa J., Tiemann J., Eckermann F., Wietfeld C., Katkov M., Huba S., Ureña J.M. (2019). A realistic evaluation of indoor robot position tracking systems: The IPIN 2016 competition experience. Measurement.

[12] Santos E.R.S., Azpurua H., Rezeck P.A.F., Corrêa M.F.S., Vieira M.A.M., Freitas G.M., Macharet D.G. (2020). Localization using ultra wideband and IEEE 802.15.4 radios with nonlinear Bayesian filters: A comparative study. J. Intell. Robotic Syst..

[13] Anderson G., Thompson J. (2020). The Economic Implications of Implementing Ultra-Wideband Technology in Industrial Settings. Econ. Ind. Tech. Rev..

[14] Patel A., Singh S. (2021). A Comparative Study of RF Interference Effects on UWB and GPS Technologies. J. Commun. Tech..

[15] Zhu X., Yi J., Cheng J., He L. (2020). Adapted error map-based mobile robot UWB indoor positioning. IEEE Trans. Instrum. Meas..

[16] Luo C., Li W., Fan X., Yang H., Ni J., Zhang X., Xin G., Shi P. (2017). Positioning technology of mobile vehicle using self-repairing heterogeneous sensor networks. J. Netw. Comput. Appl..

[17] Robertson T., Carter H. (2020). Operational Efficiency Enhancements with UWB in Autonomous Industrial Trucks. Ind. Log. Rev..

[18] Vasilyev P., Pearson S., El-Gohary M., Aboy M., McNames J. (2017). Inertial and time-of-arrival ranging sensor fusion. Gait Posture..

[19] Ding G., Lu H., Bai J., Qin X. Development of a high precision UWB/vision-based AGV and control system. Proceedings of the 5th International Conference on Control and Robotics Engineering (ICCRE).

[20] Benini A., Mancini A., Longhi S. (2013). An IMU/UWB/vision-based extended Kalman filter for mini-UAV localization in indoor environment using 802.15.4a wireless sensor network. J. Intell. Robotic Syst..

[21] An X., Zhao S., Cui X., Shi Q., Lu M. (2020). Distributed multi-antenna positioning for automatic-guided vehicle. Sensors.

[22] Wiebking L., Vossiek M., Reindl L., Christmann M., Mastela D. Precise local positioning radar with implemented extended Kalman filter. Proceedings of the European Conference on Wireless Technology.

[23] Chu Y., Ganz A. A UWB-based 3D location system for indoor environments. Proceedings of the 2nd International Conference on Broadband Networks.

[24] Mastela D., Reindl L., Wiebking L., Kawalkiewicz M., Zander T. Angle tracking using FMCW radar-based localization system. Proceedings of the International Radar Symposium.

[25] O’Neil M., Jacobs L. (2019). System Integration Strategies for UWB in Industrial Autonomous Systems. Sys. Eng. J..

[26] Kim Y., Cho J. (2021). The Role of UWB Technology in the Future of Industrial Automation. Future Ind. Tech. J..

[27] Lee A., Johnson R. (2022). Testing and Analysis of UWB Systems Under Various Industrial Conditions. J. Ind. Tests..

[28] Morgan C., Patel R. (2020). Safety Implications of Autonomous Vehicles: The Potential of UWB Technology. Saf. Sci. J..

[29] Nash B., Kramer F. (2019). High Data Transmission Rates with UWB: Benefits for Industrial Applications. Comm. Tech. Mag..

[30] Edwards S., Lin T. (2018). Power Management in UWB Systems for Efficient Industrial Applications. Energy Manag. J..

[31] Tragas P., Kalis A., Papadias C., Ellinger F., Eickhoff R., Ussmuller T., Mosshammer M., Huemer A., Dabek D., Doumenis A. RESOLUTION: Reconfigurable systems for mobile local communication and positioning. Proceedings of the 16th IST Mobile and Wireless Communications Summit.

[32] Ellinger F., Eickhoff R., Ziroff A., Hütner J., Gierlich R., Carls J., Böck G. European project RESOLUTION-local positioning systems based on novel FMCW radar. Proceedings of the IEEE MTT-S International Microwave Symposium Digest.

[33] Greene J.H., Matthews P.L. (2022). Real-world Application of UWB in Industrial AV: A Case Study. Case Stud. Ind. App..

[34] Black T., White S. (2021). Advances in UWB Technology for Precise Positioning in Industrial Environments. Adv. Tech. J..

[35] Röhrig C., Spieker S. Tracking of transport vehicles for warehouse management using a wireless sensor network. Proceedings of the IEEE/RSJ International Conference on Intelligent Robots and Systems.

[36] Adler G., Marks R. (2019). Environmental Challenges in LIDAR and UWB Operations. Env. Res. J..

[37] Tate K., Lew H. (2020). Cost Analysis of Deploying UWB Technologies in Existing Industrial Infrastructures. Fin. Rev. Ind. Tech..

[38] Liu L., Manli E., Wang Z., Zhou M. A 3D self-positioning method for wireless sensor nodes based on linear FMCW and TFDA. Proceedings of the IEEE International Conference on Systems, Man and Cybernetics.

[39] Cho H., Lee C.-W., Ban S.-J., Kim S.-W. (2010). An enhanced positioning scheme for chirp spread spectrum ranging. Expert Syst. Appl..

[40] Liu L., Manli E. Improve the positioning accuracy for wireless sensor nodes based on TFDA and TFOA using data fusion. Proceedings of the International Conference on Network, Sensor and Control (ICNSC).

[41] Zhou Y., Law C.L., Chin F. (2010). Construction of local anchor map for indoor position measurement system. IEEE Trans. Instrum. Meas..

[42] Zamora-Cadenas L., Velez I., Sierra-Garcia J.E. (2021). UWB-Based Safety System for Autonomous Guided Vehicles Without Hardware on the Infrastructure. IEEE Access.

[43] Kang D., Namgoong Y., Yang S., Choi S., Shin Y. A simple asynchronous UWB position location algorithm based on single round-trip transmission. Proceedings of the 8th International Conference on Advanced Communication Technology.

[44] Nam Y., Lee H., Kim J., Park K. Two-way ranging algorithms using estimated frequency offsets in WPAN and WBAN. Proceedings of the 3rd International Conference on Convergence and Hybrid Information Technology.

[45] Arrue N., Losada M., Zamora-Cadenas L., Jimenez-Irastorza A., Velez I. Design of an IR-UWB indoor localization system based on a novel RTT ranging estimator. Proceedings of the 1st International Conference on Sensor Device Technologies and Applications.

[46] D’Amico A.A., Taponecco L., Mengali U. (2013). Ultra-wideband TOA estimation in the presence of clock frequency offset. IEEE Trans. Wirel. Commun..

[47] Sharma S., Bhatia V., Gupta A. (2019). Joint symbol and ToA estimation for iterative transmitted reference pulse cluster UWB system. IEEE Syst. J..

[48] Joung J., Jung S., Chung S., Jeong E. (2019). CNN-based TxRx distance estimation for UWB system localization. Electron. Lett..

[49] Karapistoli E., Pavlidou F., Gragopoulos I., Tsetsinas I. (2010). An overview of the IEEE 802.15.4a Standard. IEEE Commun. Mag..

[50] Alari A., Al-Salman A., Alsaleh M., Alnafessah A., Al-Hadhrami S., Al-Ammar M., Al-Khalifa H. (2016). Ultra wideband indoor positioning technologies: Analysis and recent advances. Sensors.

[51] Muthukrishnan K., Hazas M. Position estimation from UWB pseudorange and angle-of-arrival: A comparison of non-linear regression and Kalman filtering. Proceedings of the 4th International Symposium on Location and Context Awareness (LoCA).

[52] Chen Y.-Y., Huang S.-P., Wu T.-W., Tsai W.-T., Liou C.-Y., Mao S.-G. (2020). UWB System for Indoor Positioning and Tracking with Arbitrary Target Orientation, Optimal Anchor Location, and Adaptive NLOS Mitigation. IEEE Trans. Veh. Technol..

[53] Basnayake C., Haas C., Ridenour J., Young M., Zemp R., Jayakody J., Samarakoon S. (2020). Ultra-Wideband Positioning Sensor with Application to an Autonomous Ultraviolet-C Disinfection Vehicle. Sensors.

[54] Yang K., An J., Bu X., Sun G. (2010). Constrained Total Least-Squares Location Algorithm Using Time-Difference-of-Arrival Measurements. IEEE Trans. Veh. Technol..

[55] Li A., Luan F. An Improved Localization Algorithm Based on CHAN with High Positioning Accuracy in NLOS-WGN Environment. Proceedings of the 2018 10th International Conference on Intelligent Human-Machine Systems and Cybernetics (IHMSC).

[56] Cheng Y., Zhou T. UWB Indoor Positioning Algorithm Based on TDOA Technology. Proceedings of the 2019 10th International Conference on Information Technology in Medicine and Education (ITME).

[57] Li L., Liu Z. Analysis of TDOA Algorithm about Rapid Moving Target with UWB Tag. Proceedings of the 2017 9th International Conference on Intelligent Human-Machine Systems and Cybernetics (IHMSC).

[58] Baidoo-Williams H.E., Dasgupta S., Mudumbai R., Bai E. (2013). On the Gradient Descent Localization of Radioactive Sources. IEEE Signal Process. Lett..

[59] Yağmur N., Alagöz B.B. Comparison of Solutions of Numerical Gradient Descent Method and Continuous Time Gradient Descent Dynamics and Lyapunov Stability. Proceedings of the 2019 27th Signal Processing and Communications Applications Conference (SIU).

[60] Zhang A., Lipton Z., Li M., Smola A. Dive Into Deep Learning. http://www.d2l.ai.

[61] Smith G.L., Schmidt S.F., McGee L.A. (1962). Application of Statistical Filter Theory to the Optimal Estimation of Position and Velocity on Board a Circumlunar Vehicle.

[62] Kalman R.E. (1960). A New Approach to Linear Filtering and Prediction Problems. J. Basic Eng..

[63] Welch G., Bishop G. (1995). An Introduction to the Kalman Filter.

[64] Odelson B.J., Rajamani M.R., Rawlings J.B. (2006). A new autocovariance least-squares method for estimating noise covariances. Automatica.

[65] Decawave DW1000 User Manual Version 2.18. https://www.decawave.com/dw1000/usermanual/.

[66] Ridol M., Van De Velde S., Steendam H., De Poorter E. (2018). Analysis of the scalability of UWB indoor localization solutions for high user densities. Sensors.

